# Supplementary material for: Ionitronic manipulation of current-induced domain wall motion in synthetic antiferromagnets
Source: Nat Commun. 2021 Aug 18;12:5002. doi: 10.1038/s41467-021-25292-1 (PMC8373979; doi:10.1038/s41467-021-25292-1)
Supplement: Supplementary file 1 — Supplementary Information [file 41467_2021_25292_MOESM1_ESM.pdf]

Supplementary Information for

Ionitronic manipulation of current-induced domain wall motion in synthetic antiferromagnets

Yicheng Guan[1,2], Xilin Zhou[1,2], Fan Li[1,2], Tianping Ma[1], See-Hun Yang[1], Stuart Parkin[1,*]

[1]Max Planck Institute for Microstructure Physics, 06120 Halle, Germany

[2]These authors contributed equally: Yicheng Guan, Xilin Zhou, Fan Li

*e-mail: stuart.parkin@mpi-halle.mpg.de

Supplementary Notes

Supplementary Note 1| 1-D analytical model for current-induced domain wall motion in synthetic antiferromagnetic structures

As was discussed earlier[1], the domain wall (DW) magnetization dynamics of each magnetic sub-layer in a synthetic antiferromagnetic (SAF) structure can be described by the following Landau-Lifshitz-Gilbert (LLG) equations:

$$\frac{\partial \mathbf{M}_i}{\partial t} = -\gamma \mathbf{M}_i \times \mathbf{H}_i^{\text{eff}} + \frac{\alpha_i}{m_i}\mathbf{M}_i \times \frac{\partial \mathbf{M}_i}{\partial t} - b_{\text{J}}^i \mathbf{M}_i \times \mathbf{M}_i \times \frac{\partial \mathbf{M}_i}{\partial t} - \beta_i b_{\text{J}}^i \mathbf{M}_i \times \frac{\partial \mathbf{M}_i}{\partial x} + H_{\text{she}}^i \mathbf{M}_i \times \mathbf{M}_i \times \hat{y}$$

$\mathbf{M}_i(\mathbf{r}, t)$ is the local magnetization vector, where i = U and L correspond to the upper magnetic sub-layer, and lower magnetic sub-layer, respectively. The first two terms in each equation are the field-like and damping-like torques, in the presence of an external field, $\mathbf{H}_i^{\text{eff}}$; the third and fourth terms are the Spin-Transfer Torque (STT) from polarized current injection, and the last term is the Spin-Orbit Torque (SOT) exerted via spin currents generated via the spin Hall effect from the injected current in the Pt underlayer. Here γ is the gyromagnetic ratio; α_i is the Gilbert damping parameter and β_i is the non-adiabatic parameter of the STT, for the corresponding ferromagnetic layer i; $b_{\text{J}}^i = J\frac{\mu_B P_i}{e M_s}$, where μ_B is the Bohr magneton, e is the electron's charge and P_i is the spin polarization of the current within the magnetic sub-layer i and M_i is the saturation magnetization of the sub-layer i.

In addition to the distinct magnetic parameters of the two sub-layers, the effective spin Hall effect (SHE) field H_{SHE} is also different in these sub-layers, because the spin current generated from the bottom Pt layer will experience spin depolarization as it diffuses through the lower magnetic layer, and finally, across the Ru spacer layer. Thus, we can write:

$$H_{\text{SHE}}^U = H_{\text{SHE}}^L \exp(-\frac{t_L}{\lambda_L} - \frac{t_{\text{Ru}}}{\lambda_{\text{Ru}}})$$

Here, λ_{Ru} is the spin diffusion length within the Ru spacer layer, and λ_L is the spin decoherence length of the lower FM layer, t_{L} is the thickness of the lower magnetic sub-layer, and t_{Ru} is the thickness of the Ru layer.

We now focus on the effective field exerted on the system from the relationship $\mathbf{H}_i^{\text{eff}} =$

$\frac{\partial E}{\partial \mathbf{M}_i}$, where E is the the energy per unit area, which can be written as:

$$E = 2A_L t_L \frac{\sin^2\theta_L}{\Delta^2} + t_L\left(K_L^{\text{eff}} + K_L^u \cos^2\psi_L\right)\sin^2\theta_L - M_L H_L^{\text{DMI}} \cos\psi_L \sin\theta_L + 2A_U t_U \frac{\sin^2\theta_U}{\Delta^2}$$
$$+ t_U\left(K_U^{\text{eff}} + K_U^u \cos^2\psi_U\right)\sin^2\theta_U - M_U H_U^{\text{DMI}} \cos\psi_U \sin\theta_U$$
$$- 2J_{\text{ex}}[\sin\theta_L \sin\theta_U \cos(\psi_L - \psi_U) + \cos\theta_L \cos\theta_U]$$

Here, we assume an identical DW width in both the upper and lower magnetic layers, because of the strong coupling between the two ferromagnetic sub-layers. K_i^u is the DW shape anisotropy constant, K_i^{eff} is the effective uniaxial anisotropy energy, and H_i^{DMI} is the Dzyasloshinskii-Moriya Interaction (DMI) effective field. The last term in the equation above is the exchange energy. These individual terms in E correspond to the various torques exerted on the ferromagnetic layers. The additional exchange term will give rise to an exchange coupling torque which increases the DW velocity in the SAF structure significantly, as found in earlier work (Supplementary Ref. 1).

By including these various effective fields into the LLG equation, we derive the following set of equations that describe the DW motion in a SAF system:

$$\dot{q} = \frac{\alpha_L \alpha_U}{\alpha_U M_L(1+\alpha_L^2) + \alpha_L M_U(1+\alpha_U^2)}\left[-M_L\left(\frac{1}{\alpha_L} + \beta_L\right)u_L - M_U\left(\frac{1}{\alpha_U} + \beta_U\right)u_U\right.$$
$$\mp \frac{\gamma\Delta M_L}{\alpha_L}\{\frac{H_L^k}{2}\sin 2\psi_L - \frac{\pi}{2}H_L^{\text{DMI}}\sin\psi_L - \frac{2J_{\text{ex}}}{M_L}\sin(\psi_L - \psi_U) + \frac{\alpha_L \pi H_L^{SH}}{2}\cos\psi_L\}$$
$$\pm \frac{\gamma\Delta M_U}{\alpha_U}\left\{\frac{H_U^k}{2}\sin 2\psi_U - \frac{\pi}{2}H_U^{\text{DMI}}\sin\psi_U - \frac{2J_{\text{ex}}}{M_U}\sin(\psi_U - \psi_L)\right.$$
$$\left.\left. + \frac{\alpha_U \pi H_U^{SH}}{2}\cos\psi_U\right\}\right]$$

$$\dot{\psi_L} = \pm\frac{1}{\Delta}\frac{\alpha_U}{\alpha_U M_L(1+\alpha_L^2)+\alpha_L M_U(1+\alpha_U^2)}\Bigg[-M_L\left(\frac{1}{\alpha_L}+\beta_L\right)u_L - M_U\left(\frac{1}{\alpha_U}+\beta_U\right)u_U$$

$$\mp\frac{\gamma\Delta M_L}{\alpha_L}\left\{\frac{H_L^k}{2}\sin2\psi_L - \frac{\pi}{2}H_L^{\mathrm{DMI}}\sin\psi_L - \frac{2J_{\mathrm{ex}}}{M_L}\sin(\psi_L-\psi_U) + \frac{\alpha_L\pi H_L^{SH}}{2}\cos\psi_L\right\}$$

$$\pm\frac{\gamma\Delta M_U}{\alpha_U}\left\{\frac{H_U^k}{2}\sin2\psi_U - \frac{\pi}{2}H_U^{\mathrm{DMI}}\sin\psi_U - \frac{2J_{\mathrm{ex}}}{M_U}\sin(\psi_U-\psi_L)\right.$$

$$\left.+\frac{\alpha_U\pi H_U^{SH}}{2}\cos\psi_U\right\}\Bigg] + \frac{\gamma}{\alpha_L}\{\frac{H_L^k}{2}\sin2\psi_L - \frac{\pi}{2}H_L^{\mathrm{DMI}}\sin\psi_L - \frac{2J_{\mathrm{ex}}}{M_L}\sin(\psi_L-\psi_U)\}$$

$$\pm\frac{u_L}{\alpha_L\Delta}$$

$$\dot{\psi_U} = \mp\frac{1}{\Delta}\frac{\alpha_L}{\alpha_U M_L(1+\alpha_L^2)+\alpha_L M_U(1+\alpha_U^2)}\Bigg[-M_L\left(\frac{1}{\alpha_L}+\beta_L\right)u_L - M_U\left(\frac{1}{\alpha_U}+\beta_U\right)u_U$$

$$\mp\frac{\gamma\Delta M_L}{\alpha_L}\left\{\frac{H_L^k}{2}\sin2\psi_L - \frac{\pi}{2}H_L^{\mathrm{DMI}}\sin\psi_L - \frac{2J_{\mathrm{ex}}}{M_L}\sin(\psi_L-\psi_U) + \frac{\alpha_L\pi H_L^{SH}}{2}\cos\psi_L\right\}$$

$$\pm\frac{\gamma\Delta M_U}{\alpha_U}\left\{\frac{H_U^k}{2}\sin2\psi_U - \frac{\pi}{2}H_U^{\mathrm{DMI}}\sin\psi_U - \frac{2J_{\mathrm{ex}}}{M_U}\sin(\psi_U-\psi_L)\right.$$

$$\left.+\frac{\alpha_U\pi H_U^{SH}}{2}\cos\psi_U\right\}\Bigg] + \frac{\gamma}{\alpha_U}\{\frac{H_U^k}{2}\sin2\psi_U - \frac{\pi}{2}H_U^{\mathrm{DMI}}\sin\psi_U - \frac{2J_{\mathrm{ex}}}{M_U}\sin(\psi_U-\psi_L)\}$$

$$\pm\frac{u_U}{\alpha_U\Delta}$$

By deriving the steady state solution that corresponds to $\dot{\psi_L} = \dot{\psi_U} = 0$, we can calculate the current induced DW velocity. The following parameters are used for the simulation results presented in Fig. 2d of the main text: Δ= 4.3 nm, $u_U = u_L = 12$ m s^{-1}, $\alpha_U = \alpha_L = 0.1$, $\beta_U = \beta_L = 0$, $H_L^k = H_L^k = 1400$ Oe , $H_L^{\mathrm{SH}} = 380$ Oe , $H_U^{\mathrm{SH}} = 100$ Oe , $H_L^{\mathrm{DMI}} = 1100$ Oe and $H_U^{\mathrm{DMI}} = -300$ Oe, $J_{\mathrm{ex}} = -0.4$ erg cm^{-2}.

Supplementary Figures

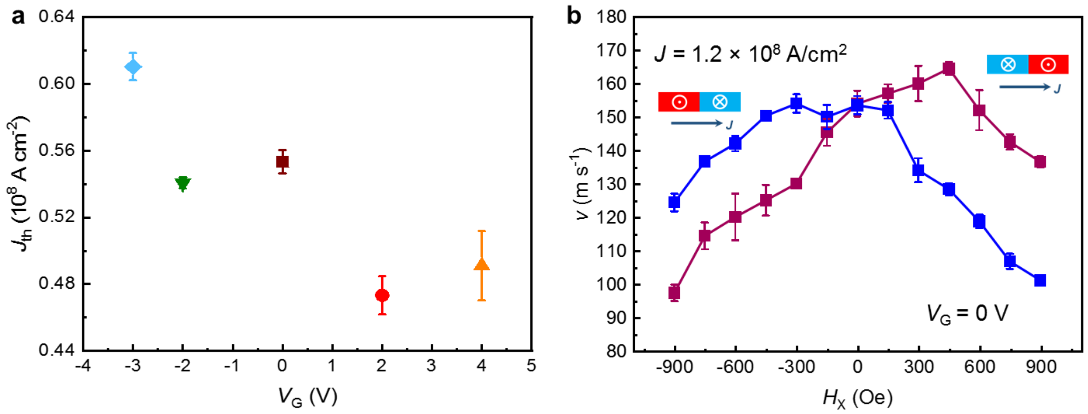

Supplementary Fig. 1| Threshold current density and longitudinal field dependence of DW velocity for two DW configurations at $V_G = 0$. a, Threshold current density, J_{th}, versus gate voltage V_G: a slight decrease in J_{th} is observed for positive V_Gs while an increase in J_{th} is found for negative V_G. **b**, The longitudinal field dependence of the DW velocity v for an up/down (filled navy square) and down/up (filled wine square) configuration, at a fixed current density (1.2×10^8 A cm^{-2}) for $V_G = 0$ (pristine state). The error bars in both figures correspond to one s.d.

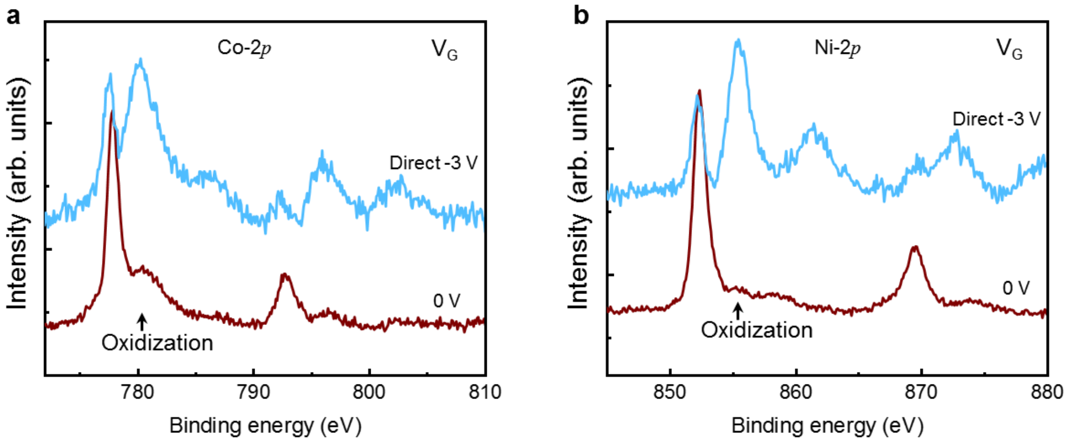

Supplementary Fig. 2| ILG induced ion migration in SAF structures. X-ray photoelectron spectra (XPS) of **a**, Co-2*p*, **b**, Ni-2*p* for SAF samples in the pristine state (wine) and after gating directly at –3 V (light blue). A much-enhanced satellite peak can be observed in both the Co-*2p* and Ni-2*p* edges, which illustrates oxidation of the Co and Ni that is induced by negative gate voltages during the ionic liquid gating process.

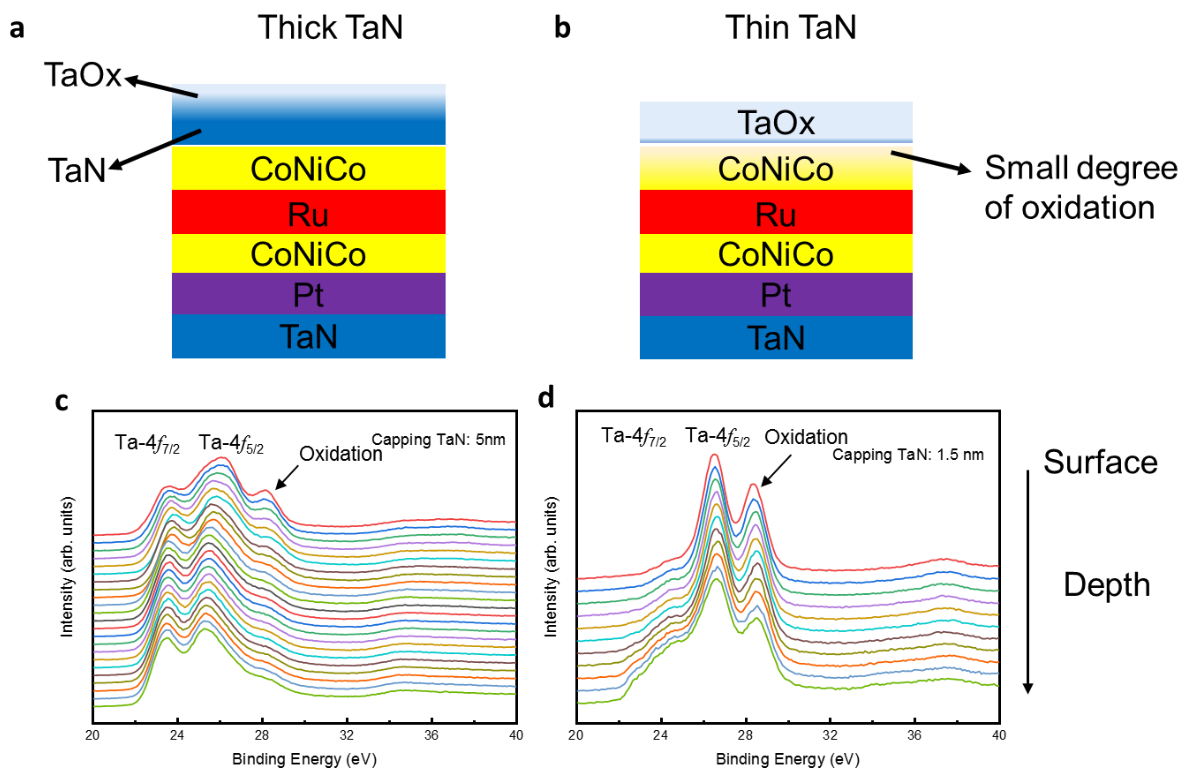

Supplementary Fig. 3| Depth profile of the Ta-4f XPS spectrum for two structures with 50 Å and 15 Å thick capping layers. The stack sequence of the films used here are TaN(20)/Pt(15)/Co(3)/Ni(7)/Co(1.5)/Ru(8.5)/Co(5)/Ni(7)/Co(1.5)/TaN(X), all units in Å. (a) and (b) are the schematic illustrations of the oxidation for the thick (X = 50) (a) and thin (X = 15) (b) TaN capping layer cases; depth profiled Ta-4*f* XPS spectra are shown in (c) and (d), correspondingly. Successive spectra correspond to increasing depth into the respective films by ~1.2 Å.

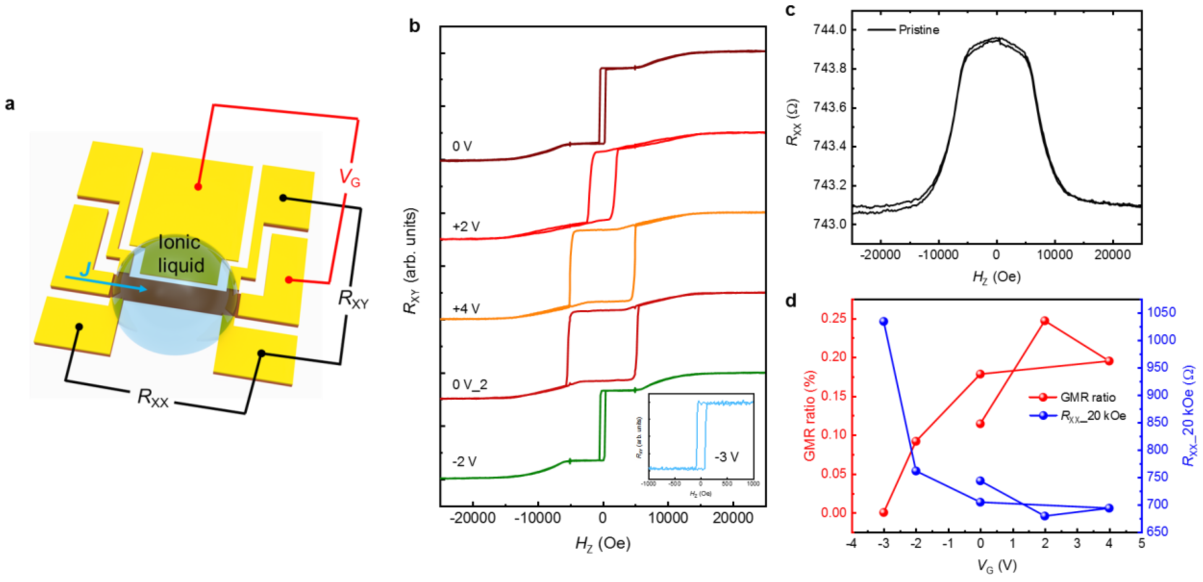

Supplementary Fig. 4| Ionic liquid gating effects on transport properties of a SAF structure. a, Sketch of Hall bar device used for transport measurements with a lateral gate electrode for ionic liquid gating. **b**, Hall resistance (R_{XY}) versus external perpendicular field at various V_G applied in the following sequence (each for 30 min): 0 V (pristine state), +2 V, +4 V, 0 V (gate voltage removed), –2 V, and –3 V. **c**, The longitudinal resistance (R_{XX}) versus external perpendicular field at pristine state. **d**, R_{XX} at a field of 20 kOe (right axis) and the giant magnetoresistance ratio (GMR ratio) (left axis) for the various V_G applied in **b**.

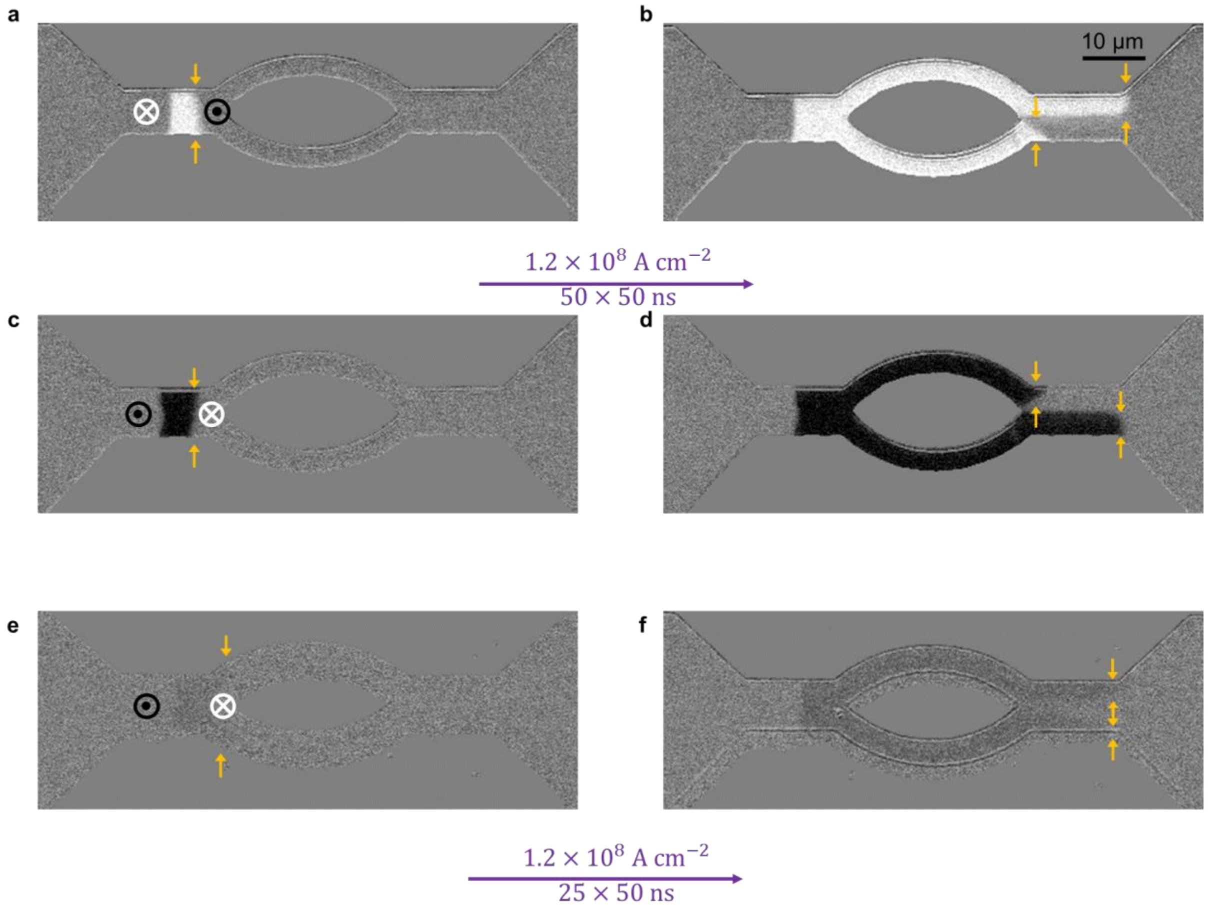

Supplementary Fig. 5| Current-induced domain wall motion through a knot device for FM and SAF structures. CIDWM through a knot device in a FM structure (with a stack sequence of TaN(20)/Pt(15)/Co(3)/Ni(7)/Co(1.5)/TaN(15), all units in Å) for up/down (**a** and **b**) and down/up (**c** and **d**) DWs. DWs with different configurations pass selectively one lane (the upper lane for the up/down DW and the lower lane for the down/up DW). In a well-compensated SAF structure (the stack corresponds to TaN(20)/Pt(15)/Co(3)/Ni(7)/Co(1.5)/Ru(8.5)/Co(5)/Ni(7)/Co(1.5)/TaN(15), all units in Å), the incoming DW can pass through both lanes (**e** and **f**).

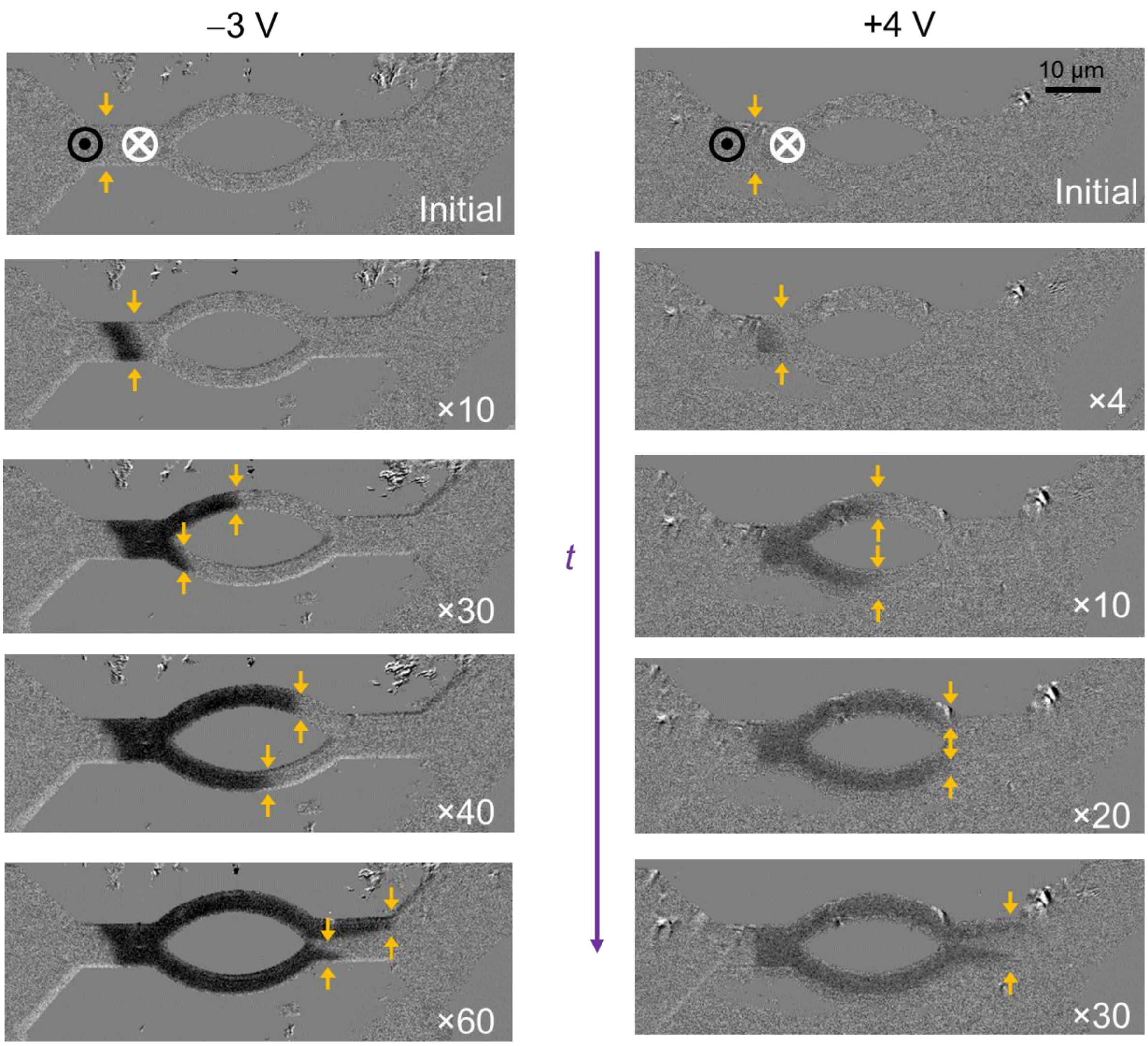

Supplementary Fig. 6| Ionic liquid gating effects on CIDWM of a SAF structure in a knot device. Current-induced up/down domain wall motion for a SAF knot device under an ionic liquid gate voltage of –3 V (left column) and +4 V (right column). Each current pulse has a current density of 1.2×10^{8} A cm^{-2} and a pulse length of 50 ns. The position of the DW(s) after a succession of current pulses is shown by yellow arrow(s). The number of current pulses in each case is indicated by the number in white at the lower right-hand corner of each image.

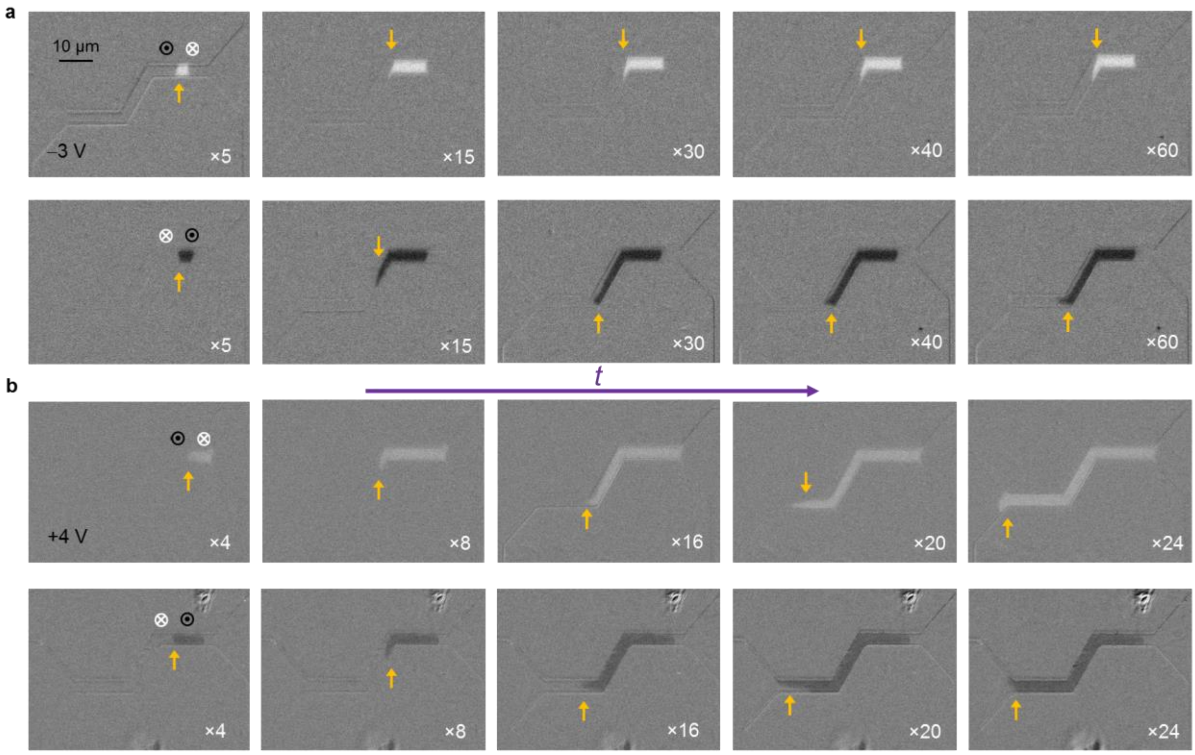

Supplementary Fig. 7| Ionic liquid gating effects on CIDWM of a SAF structure zigzag device. Current-induced up/down domain wall motion for a SAF zigzag device under an ionic liquid gate voltage of –3 V (**a**) and +4 V (**b**). Each current pulse has a current density of 1.2×10^8 A cm^{-2} and a pulse length of 30 ns. The current is injected from the right to the left. The number of current pulses in each case is given at the lower right corner of each image. The state of the initial DW is schematically illustrated in the left-most image in each row of images and the DW position is marked with the yellow arrows in each figure.

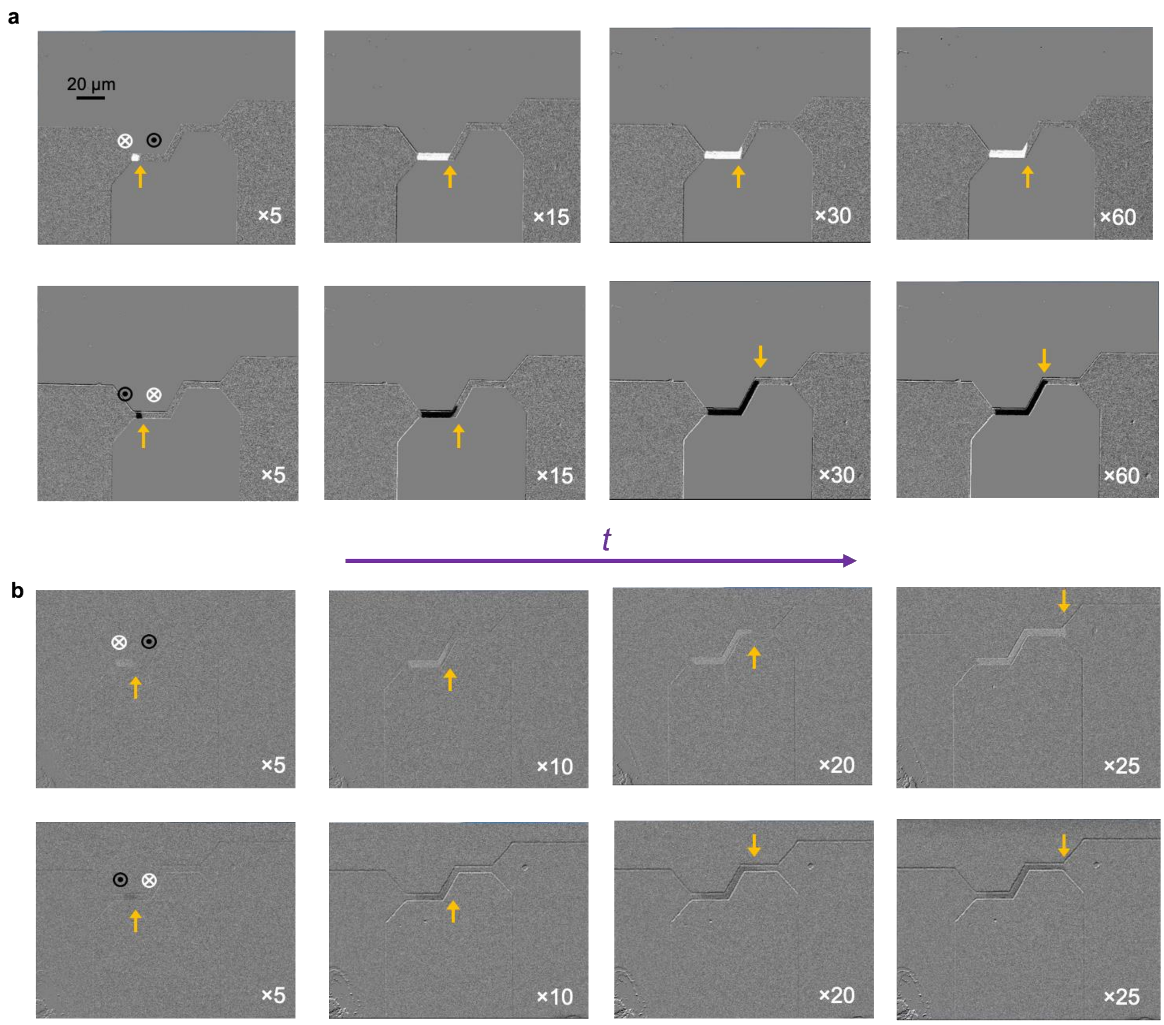

Supplementary Fig. 8| Current-induced domain wall motion in a zigzag device for FM and SAF structures. CIDWM through a zigzag device in a FM structure (with a stack sequence of TaN(20)/Pt(15)/Co(3)/Ni(7)/Co(1.5)/TaN(15), all units in Å) for up/down (**a**, first row) and down/up (**a**, second row) DWs. DWs with different configurations get stuck at the corner selectively (the second corner for the up/down DW and the first corner for the down/up DW). Each current pulse has a current density of 1.5×10^{8} A cm^{-2} and a pulse length of 30 ns. The number of injected current pulses is given at the lower right corner of each image. In a well-compensated SAF structure (the stack corresponds to TaN(20)/Pt(15)/Co(3)/Ni(7)/Co(1.5)/Ru(8.5)/Co(5)/Ni(7)/Co(1.5)/TaN(15), all units in Å), the incoming DW can pass through freely no matter the DW configurations (**b**). Each current pulse

has a current density of 1.2×10^8 A cm^{-2} and a pulse length of 30 ns. The number of current pulses is given at the lower right corner of each image. The DW position is marked with the yellow arrows in each image.

Supplementary Reference

[1] Yang, S.-H., Ryu, K.-S. & Parkin, S. S. P. Domain-wall velocities of up to 750 m s−1 driven by exchange-coupling torque in synthetic antiferromagnets. Nature Nanotechnol. 10, 221 (2015).
